# Engineering Genetic Systems for Treating Mitochondrial Diseases

**DOI:** 10.3390/pharmaceutics13060810

**Published:** 2021-05-28

**Authors:** Yoon-ha Jang, Sae Ryun Ahn, Ji-yeon Shim, Kwang-il Lim

**Affiliations:** 1 Department of Chemical and Biological Engineering, Sookmyung Women’s University, Yongsan-gu, Seoul 04310, Korea; miin_yj@sookmyung.ac.kr (Y.-h.J.); 94sjy@naver.com (J.-y.S.); 2 Industry Collaboration Center, Industry-Academic Cooperation Foundation, Sookmyung Women’s University, Yongsan-gu, Seoul 04310, Korea; saeryun@sookmyung.ac.kr

**Keywords:** mitochondrial disease, gene therapy, mitochondrial DNA, heteroplasmy, mitochondrial gene delivery

## Abstract

Mitochondria are intracellular energy generators involved in various cellular processes. Therefore, mitochondrial dysfunction often leads to multiple serious diseases, including neurodegenerative and cardiovascular diseases. A better understanding of the underlying mitochondrial dysfunctions of the molecular mechanism will provide important hints on how to mitigate the symptoms of mitochondrial diseases and eventually cure them. In this review, we first summarize the key parts of the genetic processes that control the physiology and functions of mitochondria and discuss how alterations of the processes cause mitochondrial diseases. We then list up the relevant core genetic components involved in these processes and explore the mutations of the components that link to the diseases. Lastly, we discuss recent attempts to apply multiple genetic methods to alleviate and further reverse the adverse effects of the core component mutations on the physiology and functions of mitochondria.

## 1. Introduction

Mitochondria are intracellular organelles that produce cellular energy in the form of adenosine triphosphate (ATP) through oxidative phosphorylation. Mitochondria have their own genomes as circular forms of DNA. Mitochondrial DNA (mtDNA) encodes 13 messenger RNAs (mRNAs), 2 ribosomal RNAs (rRNAs), and 22 transfer RNAs (tRNAs) required for the production of 13 protein subunits of the electron transport chain that performs oxidative phosphorylation [1]. Most cells have hundreds of mitochondria containing 2–10 copies of mtDNA [2]. More than one type of mtDNA molecule coexist in a cell. The resulting mitochondrial genome sequence heterogeneity represents a state of mitochondrial heteroplasmy [3].

Hundreds of mutations at different mtDNA sites have been reported to reduce the expression of the functional gene products required for oxidative phosphorylation [4]. When the portion of mtDNAs with such mutations out of the total mtDNAs in a cell exceeds a threshold, typically ranging from 0.6 to 0.8, the corresponding cell can be in a disease state [5]. For example, when the portion of mtDNA with the m.8993T > C mutation within the MT-ATP6 gene is higher than 0.7, neurogenic muscle weakness, ataxia, and retinitis pigmentosa (NARP) syndrome can develop. A proportion of the mutant mtDNA > 0.9 links to the development of Leigh syndrome [6].

Mutations in protein-coding mitochondrial genes can cause diseases, including Leber’s hereditary optic neuropathy (LHON), mitochondrial encephalopathy, lactic acidosis, stroke-like episodes (MELAS) syndrome, and cardiomyopathy, by resulting in the production of non-functional proteins that cannot perform oxidative phosphorylation or interfere with the reaction [7,8,9]. Given the limited resources for transcription and translation in mitochondria, such mutations can also indirectly cause a reduction in the production of functional proteins involved in the phosphorylation reaction. In addition, mutations in the regulatory regions and the genes encoding rRNA and tRNA in the mtDNAs can also lead to diseases such as Alzheimer’s disease (AD), tubulointerstitial kidney disease, ataxia, maternally inherited deafness or aminoglycoside-induced deafness (DEAF), and MELAS by altering the copy number of mtDNAs and the efficiencies of transcription and translation in mitochondria [10,11,12,13]. Various products of the genes encoded in the nuclear DNA (nDNA) also actively participate in maintaining the physiology and functions of mitochondria. Therefore, we can easily expect that mutations of such genes also cause a reduction in the rates of the oxidative phosphorylation reaction, eventually inducing the disease state of ATP deficiency [14,15].

To date, multiple attempts have been made to treat the mitochondrial disease states caused by DNA mutations [16,17,18,19]. These include targeted degradation of the mtDNA with pathogenic mutations and the introduction of a corrected version of genetic components into cells to increase the production of key parts involved in ATP production of mitochondria. In this review, we first describe the key genetic components and regulations that maintain the physiology and functions of mitochondria. Next, we list the pathogenic mutations in the genetic components and the resulting diseases. Lastly, we discuss recent therapeutic approaches to treat mitochondrial dysfunction caused by the mutations.

## 2. Regulations of Expression of Mitochondrial Genes

Replication of mtDNA is needed to maintain the copy number of the genetic materials in mitochondria. The replication also dynamically affects the heteroplasmic states of mtDNAs [20,21]. The genes in mitochondrial mtDNAs are expressed within the matrix space [22]. Mitochondrial genes are transcribed to produce long polycistronic transcripts. Through processing and maturation, the long transcripts become mRNAs, rRNAs, and tRNAs. These RNAs are involved in the translation of mitochondrial proteins [15,23]. The expression level of mitochondrial genes critically affects the organelle functions. Thus, sophisticated regulations of mitochondrial gene expression are important to maintaining cellular physiology [15,24]. Many factors encoded in nDNA are also transported to mitochondria and participate in mitochondrial gene regulations [25]. In this section, we describe how the regulations of mtDNA replication and transcription affect the mitochondrial gene expression levels. In addition, we discuss the known mutations in mtDNA and nDNA that cause disease by altering mitochondrial gene expression.

### 2.1. mtDNA Replication

#### 2.1.1. Overview of mtDNA Replication

Mammalian mtDNA is a circular, double-stranded molecule composed of a heavy (H) and a light (L) strand [26]. Each strand has a replication origin and nuclear-encoded replication factors mediate the synthesis of new strands of mtDNA through interactions with regulatory elements in mitochondrial genomes [27]. Most regulatory elements are concentrated in the mtDNA control region. According to the strand displacement model for mtDNA replication, the synthesis of the H-strand DNA is initiated at the replication origin of the H-strand (O_H_) [28,29]. RNA primers required for the initiation of DNA replication are transcribed from the L-strand and then processed by RNase mitochondrial RNA processing (MRP) complex [30]. A G-quadruplex structure between nascent RNA and non-template DNA forms at the guanine-rich conserved sequence block 2, which is located downstream of the L-strand promoter. The G-quadruplex promotes premature termination of transcription [31]. Next, RNase H1 processes the 3’end of the produced RNA, making it accessible to DNA polymerase gamma (POLγ) and function as a primer for DNA synthesis [32]. The POLγ synthesizes the H-strand DNA with the help of hexameric DNA helicase (TWINKLE) and tetrameric single-stranded DNA-binding protein (SSBP1) [33,34]. TWINKLE uncoils double-stranded DNA at the replication fork. SSBP1 prevents the formation of secondary structures of the uncoiled DNA, which is a required condition for sufficient processivity of POLγ [33,34]. The synthesis of the H-strand continues until the replication machinery reaches the O_L_, which is located approximately 11 kbp downstream of O_H_. Next, the H-strand at the origin folds into a stem–loop structure containing a poly-dT stretch. The stem–loop prevents SSBP1 from binding and mitochondrial RNA polymerase (POLRMT) synthesizes short RNA primers of around 25 nucleotides (nt) from the loop region. After the short RNA synthesis, POLRMT is replaced by POLγ, and L-strand DNA synthesis is initiated from the 3’end of the RNA primer [35]. Synthesis of both DNA strands continues until two new mtDNA molecules are formed [27,36]. The mtDNA replication machinery composed of the nuclear-encoded POLγ, TWINKLE, and SSBP1 proteins, is the main player in the mtDNA replication reactions.

#### 2.1.2. Mutations of the Replication Regulatory Elements Encoded in mtDNA

Mutations in the control region of mtDNA broadly affect the mitochondrial genetic system and are associated with mitochondrial diseases such as AD, Huntington’s disease, and melanoma [37,38,39]. For example, in brain samples of AD patients, multiple mutations were identified at different sites in the control region of mtDNA. The samples harbored mutations in the control region at frequencies 63% higher than the frequencies for the control case. The frequencies of mutations in the upstream portion (within the first 100 bases) of the control region of mtDNA (approximately 1100 bases) were not different between the AD patients and the control cases. In contrast, the frequency of mutations in the central portion (between bases 101 and 570), where the regulatory elements are enriched, was clearly higher for the AD patient cases [40]. Mutations at the conserved sequence block I (CSBI) and mitochondrial transcription factor A (TFAM) binding sites are also often found in AD patients. The m.414T > G, m.414T > C, and m.477T > C mutations are representative examples.

Similarly, several studies have found additional mutations in the mtDNA control region, which are associated with other diseases, by obtaining the corresponding DNA sequences for patients [38,41]. The mutations, m.16145G > A and m.16311T > C are associated with a higher risk of stroke [11]. It was speculated that the m.16,145G > A mutation perturbs the premature termination of H-strand elongation. This speculation was based on the fact that the mutation site is located near the termination-associated sequence responsible for DNA replication termination [42]. It was also predicted that the mutation m.16311T > C can reduce the stability of the secondary structure of the local DNA region containing base 16,311 [41]. The secondary structure of the mtDNA control region affects the binding of regulatory factors involved in mtDNA replication and transcription. Thus, mutations in this region can alter mitochondrial physiology and functions [43]. On the other hand, mutations in mtDNA that include m.72T > C, m.73A > G, and m.16356T > C may decrease the progression of myocardial infarction (MI) [41]. In addition, single nucleotide polymorphisms in the control regions of mtDNA can increase (m.16069C > T, m.16126T > C, m.16189T > C, m.16519T > C, and m.16223C > T) or decrease (m.16150C > T, m.16086T > C, and m.16195T > C) the risk of Huntington’s disease [38]. Various pathogenic mutations in the control region of mtDNA are listed in the Human Mitochondrial Genome Database (MITOMAP) [44].

#### 2.1.3. Mutations of the Nuclear Genes Involved in mtDNA Replication

Similar to the mutations in the control regions of mtDNA, those in the nuclear genes that encode the factors involved in mtDNA replication can affect the physiology and functions of mitochondria [14]. In many cases, pathogenic mutations in this group of nuclear genes lead to mitochondrial diseases by reducing mtDNA replication efficiency and causing mtDNA depletion and multiple rearrangements (mostly deletions and rarely duplications) (Table 1) [45,46]. For example, mutations in the POLG gene that encodes POLγ are linked to various diseases, including progressive external ophthalmoplegia (PEO), mitochondrial recessive ataxia syndrome (MIRAS), Parkinsonism, and Alpers–Huttenlocher syndrome [47]. To determine the mechanisms by which mutations in nuclear genes cause mitochondrial dysfunction, genetic analysis of a patient with late-onset autosomal recessive PEO (arPEO) was performed. The analysis revealed two pathogenic heterozygous missense mutations in the POLG gene: c.590T > C; Phe197Ser and c.2740A > C; Thr914Pro. The Phe197Ser mutation in the 3’-5’ exonuclease domain of POLγ reduced the exonuclease and polymerase activities of the enzyme. This reduced polymerase activity can lead to a pause of mtDNA replication during L-strand synthesis, eventually resulting in multiple mtDNA deletions. The protein with the Thr914Pro mutation has no DNA-binding affinity and therefore cannot support DNA synthesis [48].

Pathogenic missense mutations were also found within four functional domains of TWINKLE: mitochondrial targeting sequence, trimase-like domain, linker region, and helicase domain. Mutations in each domain resulted in the disruption of the corresponding function required for the full activity of TWINKLE. These severely decreased functions are the causes of mitochondrial diseases such as autosomal dominant PEO (adPEO), Perrault syndrome 5, mtDNA depletion syndrome 7, and mitochondrial hepatopathy [49]. Del Dotto et al. also identified five SSBP1 mutations associated with an optic atrophy spectrum disorder by whole-exome sequencing of five unrelated families and analyzed the effects of the mutations on mtDNA maintenance. These mutations were shown to alter the stability and multimer formation of SSBP1. The SSBP1 mutations decreased the mtDNA copy number in cells by reducing the mtDNA replication rate and resulted in mitochondrial diseases. The levels of mitochondrial dysfunction varied depending on the type of mutation of the SSBP1 gene [50].

### 2.2. Mitochondrial Transcription

#### 2.2.1. Overview of mtDNA Transcription

Transcription of mtDNA is initiated by the action of three promoters in the control region: light strand promoter (LSP), heavy strand promoter 1 (HSP1), and HSP2. LSP and HSP2 are responsible for synthesizing near-genome-length transcripts that are polycistronic, containing a single mRNA and eight tRNAs, and twelve mRNAs, thirteen tRNAs, and two rRNAs, respectively. In contrast, HSP1-mediated transcription produces a relatively short transcript containing two tRNAs and two rRNAs [57]. POLRMT initiates transcription of mtDNA by interacting with the three promoters to form transcription initiation machinery with TFAM and mitochondrial transcription factor B2 (TFB2M). After binding of TFAM upstream of the transcriptional start site, TFAM anchors POLRMT to the sites upstream of LSP and HSP1 and then recruits TFB2M, which later melts the promoter DNA sequence [58]. Once the transcription is initiated, the mitochondrial transcription elongation factor (TEFM) increases the processivity of POLRMT, resulting in the production of near-genome-length transcripts [59]. The individual mRNAs and rRNAs in the polycistronic transcripts are mostly separated by tRNAs. RNase P and elaC ribonuclease Z 2 (ELAC2) mediate the endonucleolytic cleavage at the 5’ and 3’ ends of the tRNAs, respectively, and release mRNA and rRNA from the polycistronic precursor RNA [60]. The processed mRNA, tRNA, and rRNA go through their respective maturation processes [61]. The mature tRNAs and rRNAs participate in mRNA translation in mitochondria.

#### 2.2.2. Mutations of mtDNA Transcription Regulatory Elements

Mutations in the DNA sequences related to the regulation of mitochondrial transcription can lead to mitochondrial diseases [57,62]. In Section 2.1.2, we reviewed the mutations that occur in the mtDNA control region [40]. Most of these mutations are located near the LSP, which mediates the synthesis of RNA primers used for H-strand synthesis and L-strand transcription [63]. A quantitative analysis of mtDNA copy number and mitochondrial mRNAs revealed that the local mutations resulted in a 50% reduction of the number of mtDNA and a 2-fold reduction in the number of ND6 mRNA encoded in the L-strand of mtDNA [40]. In addition, Connor et al. identified m.547A > T mutations located within the HSP of mtDNA from a large family with tubulointerstitial kidney disease [64]. The patient-derived cells showed a reduced HSP transcriptional activity. The expression levels of tRNA^Phe^ and tRNA^Leu^ encoded in the H-strand were reduced compared with the level of tRNA^Gln^ as a control [64]. Regarding the mitochondrial tRNA processing, Veronika and Rita listed disease-causing mtDNA mutations with the corresponding molecular effects and associated phenotypes [61]. More than half of all the pathogenic mutations in mtDNA are in the tRNA-coding genes [65,66]. These tRNA mutations generally have detrimental effects on tRNA biogenesis, stability, and function [61]. The best-known mutation is m.3243A > G. It is located in the MT-TL1 gene encoding tRNA^Leu (UUR)^, which is linked MELAS, chronic PEO (CPEO), and maternally inherited diabetes and deafness.

#### 2.2.3. Mutations of Nuclear Genes Involved in mtDNA Transcription

Approximately 250–300 nuclear-encoding proteins would affect mitochondrial gene expression [14,61]. A previous study reported a prevalence of nDNA mutations related to adult mitochondrial disease of 2.9 per 100,000 people in Northeast England [67]. As an example of clinical manifestations associated with defects in the nuclear genes involved in mitochondrial transcription, knockout mice with disruption of the *Polrmt*, a nuclear gene, in heart tissue developed cardiomyopathy [68]. In addition, knockout mice with disruption of the *Tefm*, another nuclear gene, showed a significantly increased heart weight [69]. Haack et al. discovered pathogenic mutations in the *Elac2* gene, which is involved in mitochondrial RNA processing, through exome sequencing of five patients with infantile hypertrophic cardiomyopathy, lactic acidosis, and isolated complex I deficiency in skeletal muscle [70]. In muscle and fibroblasts of the affected individuals, accumulation of 5’-end-unprocessed mitochondrial mRNA and rRNA precursors was observed for all the genes adjacent to mitochondrial tRNAs. Several nuclear-encoded pathogenic genes associated with the processing of precursor transcripts were also listed in another review paper [61].

In this section, we describe the details of mtDNA replication and transcription and introduced examples of mitochondrial diseases that can be caused by mutations of the genetic components involved in mtDNA expression. The genetic components are encoded by both mtDNA and nDNA.

## 3. Methods to Treat Non-Ideal Mitochondrial Gene Expression

Non-ideal profile of mitochondrial gene expression can be caused by mutations in mtDNA or nuclear genes. Untreated non-ideal mitochondrial gene expression profiles are associated with various mitochondrial diseases. Multiple studies have suggested two approaches to treat this pathogenic state: reduction of the portion of mtDNAs with pathogenic mutations in cells and introduction of corrected versions of the mutated mitochondrial genes into cells. In comparison, there have been few attempts to reverse the negative effects of mutations in the nuclear genes involved in mtDNA gene expression. Therefore, in this section, we focus on recent studies targeting the treatment of non-ideal mitochondrial gene expression, which is mainly linked to mutations in mtDNAs.

### 3.1. Reduction of mtDNAs Harboring Mutations

**Figure 1:**
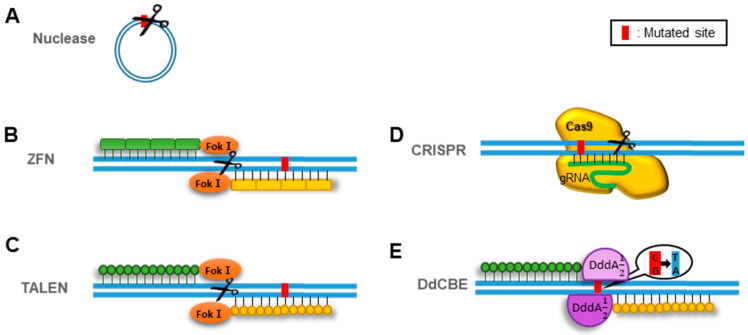
Methods of reducing mtDNAs harboring mutations to treat misregulation of mitochondrial gene expression. (**A**) Nuclease cleaves the target mutated sites. (**B**) ZFN: constructed by fusing the Fok I endonuclease with an array of zinc fingers, each having a recognition ability for a three-base DNA sequence. (**C**) TALEN: constructed by fusing Fok I with single base-recognizing domains, TALEs. (**D**) CRISPR: gRNA recognizes the mutation-including domain, and Cas9 cleaves mtDNA around the gRNA-bound site. (**E**) DdCBE: TALEs recognize the periphery of the mutation, and the two parts of DddA were fused to form the whole DddA toxin that can convert cytosine to thymidine.

Increasing the proportion of functional mtDNAs in cells by selectively degrading the mutated mtDNAs is one way to treat the state of non-ideal mitochondrial gene expression. This approach mostly employs endonucleases that can cut the mutated mtDNA sequences in a sequence-specific manner (Figure 1A). When endonucleases targeting the mutated sequences are imported into the mitochondrial matrix where mtDNAs exist, those create double-strand breaks at the mutated sites of mtDNAs. Because mitochondria lack the ability to repair double-strand breaks in DNA, the linearized DNAs were eventually degraded [71]. For example, Pst I and Sma I endonucleases transferred into the mitochondria of rodent and human cells increased cellular respiration by decreasing the proportion of mutated mtDNAs [72,73]. This approach has produced multiple successful examples of correcting the portion of functional mtDNAs in cells. However, this approach has a critical limitation in that some mutated sequences cannot be targeted because of the lack of endonucleases with the corresponding sequence specificity [74].

#### 3.1.1. Use of Zinc Fingers and Transcription Activator-Like Effectors (TALEs) as Sequence Targeting Modules

To overcome this limitation, sequence-specific DNA-binding protein domains have been fused to nucleases without sequence specificity. The first type of the domains is composed of an array of zinc fingers, each having a specificity for a three base pair DNA sequence. When multiple zinc fingers are combined, the complex can recognize longer DNA sequences (Figure 1B). The engineered mitochondria-targeted zinc-finger nuclease (mtZFN) contains a mitochondrial targeting sequence (MTS) and a synthetic endonuclease. The synthetic endonuclease was constructed by fusing the Fok I endonuclease with an array of zinc fingers [75]. This mtZFN could selectively eliminate the pathogenic mtDNAs with the m.8993T > G point mutation, which is associated with NARP and maternally inherited Leigh syndrome (MILS) [75].

Another type of DNA-binding domain is composed of an array of TALEs, each with specificity for a single DNA base. Multiple TALEs have been combined to target longer DNA sequences (Figure 1C). Mitochondria-targeted TALE nucleases (mtTALENs) have also been constructed by fusing Fok I with an array of TALEs and an MTS [76]. Two mtTALENs were used to cleave mtDNAs with the m.8344A > G mutation found in myoclonic epilepsy with ragged red fibers (MERRF) or m.13,513G > A mutation found in MELAS/Leigh [77,78].

The success of nuclease-based reduction of the portion of mutated mtDNAs highly depends on the delivery efficiency of sequence-specific nucleases into mitochondria. For enhanced delivery of nucleases viral vectors such as adeno-associated virus (AAV)-based ones have been applied. Newly expressed mtZFNs and mtTALENs after vector-mediated delivery of corresponding genes could eliminate the mutated mtDNAs and subsequently reduce the production of pathogenic factors [79,80,81]. This nuclease-based method decreases the number of mutated mtDNAs, resulting in an increase in the proportion of normal mtDNAs. The change in mtDNA heteroplasmy could persist because the long-term culture of cells expressing nucleases increases the proportion of normal mtDNAs [16,77]. To remove various mutated mtDNAs, more advanced methods for effectively constructing mtZFNs and mtTALENs are needed.

#### 3.1.2. Use of CRISPR Cas9 to Target Mutated Sequences

A new technique based on clustered regularly interspaced short palindromic repeats (CRISPR) Cas9 has been widely used to cut the mutated sites of nDNAs. The key advantage of this technique is that it can target any sequence by only altering the guide RNA (gRNA) loaded inside the Cas9 enzyme (Figure 1D). However, the application of the technique to cleave the mutated mtDNAs can be more difficult than nDNA manipulation. The Cas9 enzyme is a very large protein. It can be difficult to deliver it into the mitochondria. In addition, for transfer into mitochondria, gRNAs should contain specific sequences that form certain stem–loop structures or MTS [82,83]. There is no effective way to deliver gRNAs into mitochondria. Moreover, it is difficult to experimentally confirm the translocation of gRNA into mitochondria. Even with these difficulties, some attempts based on Cas9 and gRNA targeting the mutated mtDNAs have been successful in reducing the proportion of mutated DNAs in cells [82,83].

A CRISPR-free mtDNA editing system based on the double-stranded deaminase A (DddA) toxin, which catalyzes the deamination of cytidines of dsDNA, has been recently developed [84]. This DddA-derived cytosine base editor (DdCBE) system was engineered to split DddA into two parts at G1333 or G1397, rendering it non-toxic and inactive before binding to the target DNA (Figure 1E). The split parts of DddA were fused to MTS-linked TALE proteins that bind two adjacent DNA sites within the mitochondrial ND6 gene. To increase DNA editing efficiency one uracil glycosylase inhibitor was appended to the C-terminus of each TALE-split DddA fusion. The resultant DdCBEs efficiently mediated base editing of the mitochondrial ND6 gene in HEK293T cells by converting cytosine to thymidine [84]. If mitochondrial targeting and editing accuracy are improved, more base editing tools can be applied to correct the pathogenic mutations in mtDNA.

### 3.2. Delivery of Genetic Components to Mitochondria

Mutations of mtDNA can disrupt the genetic components involved in maintaining the physiology and functions of mitochondria, and eventually induce serious illness. Direct correction of these mutations remains technically challenging. Instead of this difficult option, the introduction of corrected versions of genetic components into cells has been suggested for the treatment of mitochondrial diseases. In this section, we describe techniques used to introduce DNA or RNA components into mitochondria to mitigate the negative effects of mutated mtDNAs.

#### 3.2.1. DNA Import into Mitochondria

##### Physical Methods

**Figure 2:**
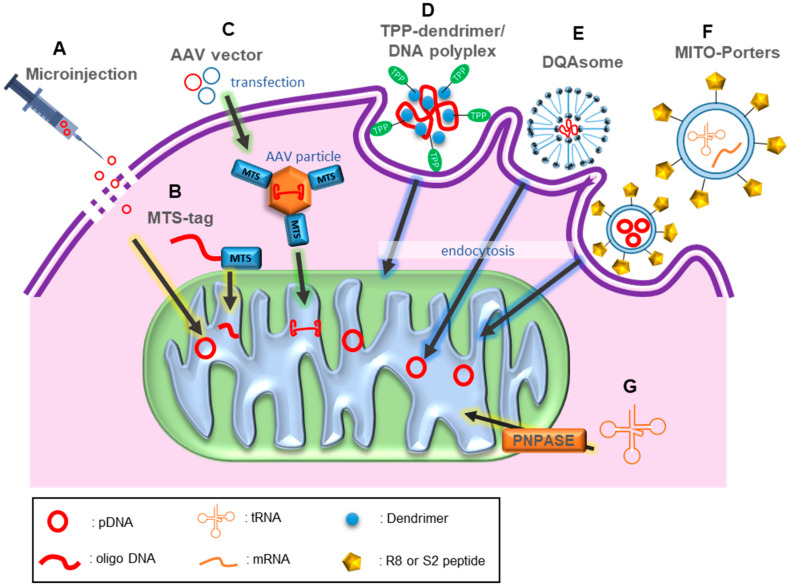
Methods for delivery of genetic components into mitochondria. (**A**) Microinjection is one of the physical methods. (B) MTS-mediated translocation can deliver DNAs into the mitochondrial matrix. (**C**) MTS-modified AAV particles can import target genes into mitochondria. (**D**) TPP-dendrimer/DNA polyplex is a dendrimer-based carrier that cannot import DNA into mitochondria but target mitochondria. Liposome-based carriers are DQAsome and MITO-Porter. (**E**) Mitochondrial expression of the gene transferred using DQAsome-mediated transfection system was confirmed. (**F**) MITO-Porters are surface-functionalized liposome-based carriers that increase transporting efficiency of target substances to mitochondria. (**G**) PNPASE can mediate the import of both small and large RNAs into mitochondria.

For nucleic acids to be delivered into mitochondria the genetic materials should pass through the plasma membrane, endosomes, and double layers of the mitochondrial membrane. Multiple physical methods have been applied to overcome these diffusion barriers [85]. One example of a physical method employed mechanical force to overcome the diffusion limitation. In one approach, a solution containing plasmid DNAs was injected into the skeletal muscle of rats using a hydrodynamic limb vein (HLV) injection system (Figure 2A). The introduced plasmid DNAs were successfully delivered into the nucleus and mitochondria without severe mitochondrial toxicity [86]. The HLV injection system promoted the flow of plasmid DNAs from vascular tissue into muscle tissue by the use of a sufficient amount of saline. The hydrodynamic forces reportedly induced temporary cell membrane openings [86]. This microinjection method is conceptually simple and direct, but its limitations include low efficiency and mechanical stress on cells [86]. In another approach, mitochondria were isolated from cells to directly introduce DNAs into them. Plasmids containing mtDNA sequences were successfully delivered into the mitochondria through electroporation that generated pores on mitochondrial membranes [87,88]. This electroporation-based method was only applied to isolated mitochondria. However, because outer mitochondria can be introduced into cells [89,90,91], one may treat cells that have defects in mtDNAs by isolating their mitochondria, transferring therapeutic DNAs into the mitochondria, and introducing the engineered mitochondria back into the cells.

##### Biological and Chemical Methods

Thirteen mitochondrial proteins are encoded in the mitochondrial genome and the remaining proteins are encoded in the nuclear genome. The nuclear-encoded proteins are synthesized in the cytoplasm of cells and are transported into mitochondria via MTS-mediated translocation (Figure 2B). MTSs are 10–80 amino acids long. They are usually located at the N-terminus of mitochondrial proteins and often contain amphiphilic helices [92,93,94]. Mitochondrial import complexes comprising translocases of the outer membrane and inner membrane recognize the MTSs of nuclear-encoded proteins and translocate the proteins into the matrix space through mitochondrial membranes. MTSs are cleaved during translocation. The mitochondrial importing system and MTS tagging have also been utilized for the delivery of nucleic acids into mitochondria [95,96]. Short double-stranded linear DNAs (17 bp or 322 bp) were successfully transported into mitochondria by MTS tagging [97]. To conjugate cargo DNA and MTS peptide, a 39 nt oligodeoxynucleotide with an amino-modified deoxythymidine in the center of its palindromic sequence was designed. The oligodeoxynucleotides form a stable loop–stem structure composed of a reactive amino group at the loop region for coupling with a unique cysteine at the C-terminus of the peptide and sticky 5’-end for cargo DNA ligation [97]. Similarly, peptide nucleic acids (PNAs)—artificial nucleic acids with an aminoethyl (pseudopeptide) backbone—can be used as carriers for gene delivery because of their DNA or RNA binding capacity and resistance to nuclease or protease attack. Furthermore, PNAs can be readily internalized into various cell lines including human fibroblasts, HeLa cells, HepG2 cells, and SY5Y cells. For mitochondrial localization, PNAs were conjugated with MTS and the PNA-MTS conjugates were translocated into the mitochondrial matrix of cultured human myoblasts in a membrane potential-dependent manner [98]. In another study, PNA linked with yeast cytochrome oxidase (COX) IV mitochondrial targeting peptide successfully mediated the transport of oligonucleotides annealed to the PNA into the mitochondrial matrix of mouse myoblasts [99]. Transferred short oligo DNA can act as a selective inhibitor for mutated mtDNA replication or can mediate the repair of mtDNA with point mutations. Furthermore, MTS conjugation to various carriers applied for general gene therapy can be a strategy to introduce large nucleic acids, such as plasmid DNA, into the mitochondrial matrix.

AAV vectors have attracted much attention in the field of gene and cell therapy because they can transduce genes into many types of cells without severe adverse effects [100,101,102]. AAV is a parvovirus having a DNA genome of approximately 4.7 kb. Its single-stranded genome contains viral replicase (*rep*) and capsid (*cap*) genes between two inverted terminal repeats (ITRs) [102]. The *rep* gene encodes four enzymatic proteins (Rep78, Rep68, Rep52, and Rep40), and the *cap* gene encodes three capsid proteins (VP1, VP2, and VP3) [102]. For applications of AAV as gene delivery vectors, the AAV protein-coding sequences between the ITRs are substituted with therapeutic gene expression cassettes, and the *rep* and *cap* genes are provided *in trans* [103]. In this way, AAV vectors carrying the mitochondrial *ND4* gene expression cassette were generated [17]. The *ND4* gene can only be translated into mitochondria because the gene uses TGA, which is recognized as a tryptophan codon rather than as a stop codon in mitochondria. An MTS was inserted into the N-terminus of the capsid VP2 sequence to guide the AAV vectors to mitochondria (Figure 2C). The cytoplasmic hybrid (cybrid) cell lines containing mitochondria with the m.11778G > A mutation in the *ND4* gene were infected with the MTS-modified AAV vectors carrying a wild-type *ND4* gene. The delivered functional *ND4* gene was expressed in the cells and their defective respiratory function was rescued. In an in vivo study, AAV vectors that carry a functional *ND4* gene and MTS were injected into the eyes of mice. Two days later, AAV vectors carrying an *ND4* gene with a mutation of Arg340His, which is known to cause visual loss in rodents, were additionally injected into the eyes that were treated with the functional *ND4* gene. The introduced functional ND4 gene prevented visual impairment in the mice, and the therapeutic effects persisted for almost the entire life span of the mice [17]. In a subsequent study, the DNAs containing the *ND4* gene that were delivered by MTS-modified AAV vectors remained episomally in the mitochondrial matrix without integration into the mitochondrial genome [104]. These results suggest a possibility that viral vectors commonly used in gene therapy can be engineered to target mitochondria by tagging MTS to viral structural proteins.

Lipophilic cations such as ethidium, tetraphenylphosphonium (TPP), triphenylmethylphosphonium (TPMP), and tetraphenylarsonium (TPA) also have mitochondria-targeting capabilities. Lipophilic ions can permeate across biological membranes composed of phospholipid bilayers, and cationic molecules can accumulate in the negatively charged mitochondrial matrix as a result of the electrochemical equilibration of ions [105]. TPP has been applied to target various molecules to mitochondria by conjugation with the molecules directly or by carrier packaging of the molecules [106,107,108,109]. As an example of TPP-mediated DNA delivery, an 11-mer PNA conjugated with TPP was taken up by mitochondria within 143 B osteosarcoma cells [110]. The sequence of the PNA oligomer was designed to be complementary to the mtDNA containing the MERRF disease-causing m.8344A > G mutation to selectively inhibit the mutated mtDNA replication in cells. In another study, TPP was conjugated to polymer-based dendrimers that can condense genetic materials [111]. Plasmid DNAs containing the genes that encode enhanced green fluorescent protein or luciferase were incubated with TPP-conjugated dendrimers to generate TPP-dendrimer/DNA polyplexes (Figure 2D). The mitochondria-targeting ability of the polyplexes was experimentally demonstrated, but the mitochondrial import of the transferred DNA was not confirmed. The data collectively indicate that MTS actively transports cargo molecules into the mitochondrial matrix by passing through the channels on the mitochondrial membrane, while TPP passively diffuses depending on the mitochondrial membrane potential.

For the passage of negatively charged mitochondrial lipid bilayer membranes, liposome-based carriers have been used to encapsulate the DNA. The DQAsome is a liposome-like aggregate formed with dequalinium. Dequalinium can accumulate in mitochondria due to the delocalized cationic charge [112,113]. As DQAsomes can bind and condense plasmid DNA, DQAsome-DNA complexes (DQAplexes) were prepared by simply mixing the DNA with DQAsomes [114]. The applicability of DQAsomes as mitochondria-targeting carriers for DNA was investigated (Figure 2E). Fluorescence imaging of live BT20 cells exposed to DQAplexes revealed the endosomal escape of DQAplexes and the liberation of plasmid DNAs from DQAplexes at the site of mitochondria [114]. In addition, Weissig et al. provided evidence that DQAplexes release DNA upon contact with the mitochondrial membrane through experiments using cardiolipin-rich liposomes mimicking mitochondrial membranes and isolated mouse liver mitochondria [113,115]. DQAsomes were used to transfect plasmid DNA containing the GFP gene designed for mitochondrial expression (mtGFP) into several mammalian cell lines [116]. Mitochondrial expression of the mtGFP gene transferred by the DQAsome-mediated transfection system was experimentally confirmed. Although the expression level was low, the result was significant in that it is one of the few methods to date that has enabled mitochondrial expression of genes introduced into live cells. Another study showed that the mitochondria-targeting characteristics of DQAsomes can be enhanced by anchoring mitochondriotropic ligands such as TPP to the liposomal phospholipid bilayer [117].

Liposome-based carriers for mitochondrial gene delivery have been improved through surface modification and lipid composition optimization. MITO-Porters developed by Yamada et al. displayed octaarginine (R8) on the surface for cellular internalization of the liposome [118]. In addition, the original lipid component, egg yolk phosphatidylcholine, was substituted by 1,2-dioleoyl-sn-glycero-3-phosphatidylethanolamine (DOPE). This lipid composition increased the fusogenic activity of liposomes, consequently allowing endosome escape of the liposome-DNA complex and fusion between the liposomal membrane and mitochondrial membrane [118] (Figure 2F). In a subsequent study, dual-function MITO-Porter (DF-MITO-Porter) was generated to improve mitochondrial drug delivery efficiency [119]. The DF-MITO-Porter comprises a mitochondria-fusogenic inner lipid envelope and an endosome-fusogenic outer lipid envelope, whose surfaces are modified with R8. The penetration efficiency of this new liposome-based carrier for mitochondria was higher than that of conventional MITO-Porter (83.3% versus 25.0%) [119]. To evaluate the delivery of cargo molecules into the mitochondrial matrix, DNaseI protein was encapsulated in DF-MITO-Porter and mtDNA levels in the mitochondrial matrix were analyzed [119,120]. After DF-MITO-Porter-mediated mitochondrial delivery of DNase I, a substantial decrease in mtDNA levels was observed compared with the conventional MITO-Porter case [119,120]. Aside from the R8 peptide, five kinds of peptides with favorable properties for mitochondrial gene transfer were used as ligands for surface modification of MITO-Porters. MITO-Porter modified with S2 peptides (Dmt-_D_-Arg-Phe-Lys-Dmt-_D_-Arg-Phe-Lys, Dmt = 2, 6-dimethyltyrosine) showed a similar mitochondrial targeting activity, but lower cytotoxicity compared to those of the one modified with R8 [121]. Using the MITO-Porters, both short oligo DNAs and plasmid DNAs were successfully transferred into mitochondria in live HeLa cells [121,122,123]. Finally, mitochondrial localization of cy5-labeled oligo DNAs was observed using confocal laser scanning microscopy [121,122]. In another study, MITO-Porter modified with cell-penetrating KALA peptides delivered plasmid DNAs containing the gene that encodes luciferase using the mitochondrial codon system [123]. The potent CMV promoter derived from cytomegalovirus induced luciferase expression. This is one of the few examples of transgene expression in mitochondria.

#### 3.2.2. RNA Import into Mitochondria

Efficient delivery of RNA into mitochondria can be an alternative to treat mitochondrial diseases caused by mutations in nDNAs or mtDNAs. Several methods to deliver RNA into mitochondria have utilized endogenous RNA import systems. Similar to mitochondrial proteins, some RNAs that function in the mitochondria are encoded and transcribed in the nucleus [124]. The pre-protein import apparatus mediates the mitochondrial uptake of cytosolic small RNAs depending on ATP. In mammals, three types of RNAs are imported into mitochondria: 5S rRNA, RNA component of RNase P (H1 RNA), and RNA component of RNase MRP (RMRP) [125]. These are all imported into mitochondria through the pre-protein import channel, and protein factors that direct the RNA translocation exist. For example, the mitochondrial ribosomal proteins MRP-L18 and rhodanese (thiosulfate sulfurtransferase) are protein factors involved in mitochondrial import of 5S rRNA [126,127]. MRP-L18 induces a conformational change of 5S rRNA by binding to the γ-domain and allows interaction between the rhodanese and the α-domain of 5S rRNA. Subsequently, the RNA-protein complex is imported into mitochondria [124].

In addition, polynucleotide phosphorylase (PNPASE) is a well-known protein that mediates the import of nuclear-encoded RNAs, including 5S rRNA, H1 RNA, and RMRP, into the mitochondrial matrix [128]. Mammalian PNPASE has an MTS at its N-terminus and recognizes a 20 nt stem–loop structure in H1 RNA. In similar, PNPASE interacts with a different 20 nt sequence forming a stem–loop structure in RMRP [129]. In a study, fusion of one of the stem–loop sequences to the 5’-end of the glyceraldehyde 3-phosphate dehydrogenase (GAPDH) RNA allowed the import of the RNA into isolated yeast mitochondria [128] (Figure 2G). The sequence forming a stem–loop structure also enabled the translocation of human mitochondrial tRNA^trp^ into isolated mouse liver mitochondria [128]. In the following study, this approach was also applied to transfer nuclear-encoded tRNAs into mitochondria to mitigate the disease state of MERRF caused by the m.8344A > G mutation in the mitochondrial tRNA^Lys^ and MELAS caused by the m.3243A > G mutation in the mitochondrial tRNA^Leu^ [130]. In addition to appending the RNA import sequence of H1 RNA to the tRNA precursor sequences, two more strategies were applied. First, the aminoacyl stems of the mitochondrial tRNA^Lys^ and tRNA^Leu^ precursors were extended by changing several unpaired ribonucleotides adjacent to the stem region to be paired to prevent cleavage of the H1 RNA import sequence from the tRNA precursors. Second, for RNA localization to the mitochondrial outer membrane, the 3’-untranslated region (UTR) of transcript for mitochondrial ribosomal protein S12 (*MRPS12*) was additionally appended to the 3’-end of the extended tRNA precursors with the H1 RNA import sequence. As a result, in MERRF and MELAS cybrid cells expressing the rationally engineered mitochondrial tRNA precursors, defects in mtRNA translation and cellular respiration were rescued. Furthermore, using the interaction between PNPASE and the H1 RNA import sequence, the import of relatively large mRNAs as well as tRNA precursors into the mitochondrial matrix was also confirmed. A mitochondrial-encoded mouse *COX2* gene was linked with the H1 RNA import sequence. The resultant DNA construct was introduced into HeLa cells via transient transfection. RT-PCR was used to examine the import of RNA transcribed from the introduced DNA. Only the genes fused to the H1 RNA import sequences were imported into the mitochondrial matrix [130]. PNPASE, which guides cytosolic small RNAs into mitochondria in cells, can mediate the import of both small and large RNAs. As this approach takes advantage of the proteins originally existing in mammalian cells, it is expected to have lower cytotoxicity than other approaches using artificial carriers.

Currently, it is known that tRNA^Gln^ is the only tRNA that translocates from the cytosol to mitochondria in mammalian cells [131]. In contrast, in yeast more types of tRNA can be imported into mitochondria. Therefore, there have been attempts to treat human mitochondrial dysfunction caused by mutations in tRNAs with yeast tRNAs. In a study, it was experimentally confirmed that several yeast tRNA^Lys^ derivatives can be internalized into isolated human mitochondria through the protein import channels [125]. In addition, mammalian cytosolic aminoacyl-tRNA synthetase mediated aminoacylation of the yeast tRNA^Lys^ efficiently as yeast aminoacyl-tRNA synthetase did [125]. Based on this result, Kolesnikova et al. suggested a therapeutic strategy to treat MERRF syndrome caused by mutations in mtDNA encoding the tRNA^Lys^ [18]. Plasmid DNA containing the yeast tRNA^Lys (CUU)^ sequence was introduced into cybrid cells carrying the m.8344A > G mutation in mtDNA and MERRF patient-derived fibroblasts via transient transfection. Expression of the yeast tRNA^Lys^ partially rescued defects in mitochondrial functions such as mitochondrial translation, cellular respiration, and membrane potential generation [18]. This method is based on allotropic expression, in which DNAs encoding mitochondrial genes are transferred to the nucleus and the resultant gene products in the cytoplasm are re-localized to mitochondria.

In contrast, mtRNAs could be directly delivered to mitochondria by using the aforementioned liposome-based MITO-Porter [132]. Wild-type mitochondrial tRNA^Phe^ precursors produced via an in vitro transcription system with T7 RNA polymerase were complexed with protamine and encapsulated by MITO-Porter for mitochondrial localization. The resulting MITO-Porter complexes were introduced into fibroblasts derived from a patient carrying the m.625G > A mutation in the mitochondrial gene encoding tRNA^Phe^. To confirm the effects of MITO-Porter-mediated RNA delivery, the fractions of mutant tRNA^Phe^ in the mitochondria of the control and treated cells were evaluated. The fraction of the mutant tRNA^Phe^ in the mitochondria of the untreated control cells was around 0.8. After transfection of MITO-Porter encapsulating wild-type tRNA^Phe^ that was modified with tRK1 sequence having the mitochondrial import activity, the fraction of the mutant was decreased to around 0.2. In another attempt, MITO-Porter was used to deliver an antisense RNA oligonucleotide (ASO) of 46 nt targeting the *COX2* gene [133]. The 5’-end of the ASO was modified with a Darm sequence that binds to the RNA import complex protein for mitochondrial targeting activity. Darm-ASOs were complexed with polyethyleneimine (PEI) polycation, and the complexes were encapsulated in MITO-Porter. The MITO-Porter enabled the RNA/PEI complex to reach the intermembrane space of mitochondria in HeLa cells, and the ASOs were imported into the mitochondrial matrix via the Darm import machinery. In cells transfected with Darm-modified ASOs, the expression level of COX2 protein was decreased, inhibiting ATP production, whereas ASOs without the Darm sequence did not affect the COX2 level [133]. This finding suggests that the expression of specific mitochondrial genes can be regulated through gene silencing by mitochondrial delivery of ASOs.

MITO-Porter can also mediate the delivery of relatively large mRNA [19]. The synthesis of ND3 mRNA was achieved by T7 promoter-driven in vitro transcription. ND3 mRNA formed a complex with positively charged protamine for encapsulation into MITO-Porter nanoparticles. MITO-Porters carrying normal ND3 mRNAs were introduced into fibroblasts from a Leigh syndrome patient harboring the m.10158T > C mutation in the *ND3* gene. To confirm the mitochondrial delivery of ND3, the proportion of mutant ND3 mRNA in the mitochondria of fibroblasts, which were obtained from a patient with Leigh syndrome caused by the m.10158T > C mutation in mtDNA, and the oxygen consumption rate indicating the mitochondrial respiratory activities were both evaluated. Compared with non-treated cells showing the mutant proportion of approximately 0.8, the mutant proportion of the treated cells was significantly decreased to 0.1 in a dose-dependent manner. In addition, the maximal oxygen consumption rate of the cells treated with the MITO-Porter carrying normal ND3 mRNAs was about 1.46-fold higher than that of the untreated cells. These data indicate that mitochondrial respiration activity can be improved by the introduction of normal mRNAs into mitochondria [19]. The use of MITO-Porters can be a potential therapeutic strategy for mitochondrial diseases caused by mutations in mtDNA because of their ability to encapsulate various forms of nucleic acids and pass through biological membranes, if the basal cytotoxicity of the liposome-based carriers can be overcome.

## 4. Concluding Remarks

Mutations in mitochondrial genomes can cause diseases involving malfunction of various organs, including the brain, nerves, eyes, and heart, due to impaired energy production. Correction of the mutations or introduction of functional genetic components into mitochondria can be a reasonable strategy to cure mitochondrial diseases. However, two separate lipid membranes of mitochondria do not allow easy access of therapeutic genetic components to the matrix space where the mitochondrial genetic system mainly operates. The mixed and heterogeneous nature of mitochondrial DNAs in a cell complicates the design and applications of the gene therapy strategy. In this review, we introduced multiple aspects of basic regulations of mitochondrial DNA replication and transcription. Mutations of the genetic components that affect the physiology and functions of mitochondria and linkages between the mutations and diseases have been also discussed. Various studies targeted to reduce the number of mutated DNAs in mitochondria and enhance the delivery system for the introduction of therapeutic genetic components into mitochondria. To reach the goal of treating and eventually curing mitochondrial diseases, various approaches, employing physical forces, newly constructed chemical complexes, and recombinant proteins, have been applied. Partial success in the studies encourages future ambitious attempts to effectively cure mitochondrial disease. A better understanding of molecular and genetic mechanisms involved in mitochondrial diseases and the fusion of multidisciplinary approaches will further fuel the advances in mitochondrial gene therapy.

## Figures and Tables

**Table 1:** Diseases caused by mutations of the proteins regulating mtDNA replication.

Gene	Function	Disease	References
POLG	POLγ catalytic subunit	MIRAS, Parkinsonism, AHS, MCHS, MEMSA, ANS, ad/ar PEO, male infertility, testicular cancer	[47,51]
POLG2	POLγ accessory subunit	adPEO	[52]
TWINKLE	mtDNA helicase	PEO, hepatopathy, spinocerebellar ataxia, epileptic encephalopathy	[49]
RNASE H1	Endoribonuclease of the RNA-DNA hybrid	CPEO, exercise intolerance	[53]
SSBP1	Subunit of ssDNA-binding complex	optic atrophy	[50]
MGME1	Metal dependent ssDNA exonuclease	recessive multi-systemic mitochondrial disorder	[54]
TOP3A	Topoisomerase	CPEO plus syndrome	[55]
TFAM	Transcription factor	neonatal failure	[56]

Abbreviations: MIRAS, Mitochondrial recessive ataxia syndrome; AHS, Alpers–Huttenlocher syndrome; MCHS, myocerebrohepatopathy spectrum; MEMSA, myopathy sensory ataxia; ANS, ataxia neuropathy spectrum; CPEO, chronic progressive external ophthalmoplegia.

## Abbreviation

ATP	adenosine triphosphate
AAV	adeno-associated virus
AD	Alzheimer’s disease
adPEO	autosomal dominant PEO
AHS	Alpers–Huttenlocher syndrome
ANS	ataxia neuropathy spectrum
arPEO	autosomal recessive PEO
ASO	antisense RNA oligonucleotide
cap	capsid
COX	cytochrome oxidase
CPEO	chronic PEO
CPEO	chronic progressive external ophthalmoplegia
CRISPR	clustered regularly interspaced short palindromic repeats
CSBI	conserved sequence block I
DdCBE	DddA-derived cytosine base editor
DddA	double-stranded deaminase A
DEAF	deafness or aminoglycoside-induced deafness
DF-MITO-Porter	dual function MITO-Porter
DOPE	1,2-dioleoyl-sn-glycero-3-phosphatidylethanolamine
DQAplexes	DQAsome-DNA complexes
ELAC2	elaC ribonuclease Z 2
GAPDH	glyceraldehyde 3-phosphate dehydrogenase
gRNA	guide RNA
HLV	hydrodynamic limb vein
HSP1	heavy strand promoter 1
ITRs	inverted terminal repeats
LHON	Leber’s hereditary optic neuropathy
LSP	light strand promoter
MCHS	myocerebrohepatopathy spectrum
MELAS	mitochondrial encephalopathy, lactic acidosis, stroke-like episodes
MEMSA	myopathy sensory ataxia
MERRF	myoclonic epilepsy with ragged red fibers
MI	myocardial infarction
MILS	maternally inherited Leigh syndrome
MIRAS	Mitochondrial recessive ataxia syndrome
MIRAS	mitochondrial recessive ataxia syndrome
mRNAs	messenger RNAs
MRP	mitochondrial RNA processing
MRPS12	mitochondrial ribosomal protein S12
mtDNA	mitochondrial DNA
MTS	mitochondrial targeting sequence (
mtTALENs	mitochondria-targeted TALE nucleases
mtZFN	mitochondria-targeted zinc-finger nuclease
NARP	neurogenic muscle weakness, ataxia, and retinitis pigmentosa
nDNA	nuclear DNA
nt	nucleotides
O_H_	origin of the H-strand
PEI	polyethyleneimine
PEO	progressive external ophthalmoplegia
PNAs	peptide nucleic acids
PNPASE	polynucleotide phosphorylase
POLRMT	mitochondrial RNA polymerase
POLγ	DNA polymerase gamma
rep	replicase
RMRP	RNase MRP
rRNAs	ribosomal RNAs
SSBP1	single-stranded DNA-binding protein
TALEs	transcription activator-like effectors
TEFM	mitochondrial transcription elongation factor
TFAM	mitochondrial transcription factor A
TFB2M	mitochondrial transcription factor B2
TPA	tetraphenylarsonium
TPMP	triphenylmethylphosphonium
TPP	tetraphenylphosphonium
tRNAs	transfer RNAs
UTR	untranslated region
